# CRISPR-Cas13a SHERLOCK assay for rapid and sensitive detection of chikungunya virus

**DOI:** 10.1128/spectrum.02298-25

**Published:** 2025-12-19

**Authors:** Niracha Athipanyasilp, Suwanna Saowpak, Chutikarn Chaimayo, Nasikarn Angkasekwinai, Archiraya Pattama, Artittaya Athipanyasilp, Maturada Patchsung, Kanokpol Aphicho, Chayasith Uttamapinant, Navin Horthongkham

**Affiliations:** 1 Department of Microbiology, Faculty of Medicine, Siriraj Hospital, Mahidol University 26685, Bangkok, Thailand; 2 Department of Medicine, Faculty of Medicine, Siriraj Hospital, Mahidol University 26685, Bangkok, Thailand; 3 School of Biomolecular Science & Engineering, Vidyasirimedhi Institute of Science and Technology 423058, Rayong, Thailand; National Chung Hsing University, Taichung, Taiwan

**Keywords:** chikungunya virus, CRISPR, SHERLOCK, recombinase polymerase amplification (RPA), rapid diagnosis

## Abstract

Chikungunya virus (CHIKV), a major cause of acute febrile illness and joint pain, remains a significant public health threat in tropical regions. Rapid and accurate detection is essential for timely clinical management and outbreak control, particularly in resource-limited settings where real-time PCR (RT-qPCR) is often impractical. We developed and validated a SHERLOCK assay coupled with recombinase polymerase amplification for CHIKV RNA detection. Analytical performance was assessed by determining the limit of detection (LOD), cross-reactivity, clinical sensitivity and specificity, and predictive values. The assay achieved an LOD of 215 copies/reaction with no cross-reactivity against other alphaviruses or flaviviruses. Clinical testing of 146 plasma samples showed a sensitivity and specificity of 94.52% and 100% with lateral-flow readout and 97.26% and 100% with fluorescence readout, respectively. This study establishes a promising CRISPR-Cas13a-based SHERLOCK platform for CHIKV detection, demonstrating high analytical performance, rapid turnaround time, and potential for future adaptation to resource-limited settings.

**IMPORTANCE:**

Early and accurate detection of chikungunya virus (CHIKV) is critical for outbreak control, especially in resource-limited settings, where real-time PCR is not feasible. This study demonstrates that the CRISPR-Cas13a-based SHERLOCK platform, combined with RPA, achieves high diagnostic accuracy and a low detection limit, comparable to RT-qPCR. The assay’s rapid turnaround time and simple lateral-flow readout make it a promising tool for point-of-care diagnostics during CHIKV outbreaks, potentially improving disease surveillance and clinical decision-making.

## INTRODUCTION

The chikungunya virus (CHIKV) belongs to the family *Togaviridae* (genus, *Alphavirus*) and was first isolated in 1952 from patient-infected serum in Tanzania (1, 2). CHIKV is found extensively throughout tropical and subtropical areas and transmitted to humans by *Aedes* mosquitoes. Since the discovery of CHIKV, there have been periodic outbreaks of this virus in Africa, Asia, and the Indian Ocean region. CHIKV strains can be classified into nine lineages: Asian urban, Asian urban-America, South America, Middle Africa, Indian Ocean, East Africa, Africa and Asia, East/Central/South Africa, and West Africa (3). CHIKV outbreaks were observed in Thailand in 1960 and were of the Asian genotype (4). The next outbreak in Thailand occurred in the country’s southern region in 2008 and 2009, with morbidity rates of 3.95 and 82.03/100,000 population, respectively (5). The East/Central/South African genotype was reported in this outbreak, with a mutation at A226V of the E1 protein (6). More outbreaks in Thailand occurred in 2018 and 2019, with morbidity rates of 5.4 and 16.9/100,000 population, respectively (7, 8). The East/Central/South African genotype was still reported for these outbreaks (9). CHIKV continued spreading in Thailand during 2020, with a morbidity incidence of 16.46/100,000 population, but declined in 2021 (10). CHIKV infection includes symptoms of fever, headache, skin rash, and joint pain (11). These symptoms are similar to those of other arthropod-borne viruses, such as dengue and Zika viruses.

Rapid, reliable, and low-cost diagnostic platforms are essential to control the spread and effectively manage the treatment of viruses. The laboratory diagnosis of CHIKV relies on several methods, such as a nucleic acid test (reverse transcription polymerase chain reaction [RT-PCR]), a serological assay (enzyme-linked immunosorbent assay [ELISA]), and rapid antibody detection. RT-PCR is more sensitive and is usually used to detect the early stages of infection (12). In a resource-limited setting, RT-PCR is unsuitable because of the need for a cost-effective real-time PCR system. Loop-mediated isothermal amplification was developed to address this issue with high sensitivity and specificity while operating under constant temperature conditions, eliminating the need for thermal cycling or real-time instrumentation (13). Recently, cluster regularly interspaced short palindromic repeat (CRISPR) has been introduced to infectious diagnostic systems for detecting coronavirus disease 2019 and has received attention because of its simplicity, high sensitivity, and specificity (14, 15). The CRISPR system consists of CRISPR RNA (crRNA) and CRISPR-associated (Cas) nucleases. The crRNA is designed to recognize the target site, form a complex with Cas, and trigger cleavage activity. There are various CRISPR-Cas systems, such as Cas9, Cas12, and Cas13 variants. Cas12 and Cas13 enzymes are widely used in diagnostic tests because they possess trans-acting cleavage activity, which can be used to cleave nucleic acid reporters, generating fluorescence, electrochemical, or lateral-flow strip-compatible signals (14, 16). Specific high-sensitivity enzymatic reporter unlocking (SHERLOCK) was developed by coupling RPA with RNA-targeting Cas13, which can detect specific target sequences using a lateral-flow dipstick or fluorescent readout (16, 17). SHERLOCK has been successfully developed and can detect various viral infections, such as dengue, Zika, West Nile, yellow fever, and severe acute respiratory syndrome coronavirus 2 (SARS-CoV-2) (15, 18).

In this study, RPA coupled with a CRISPR-Cas13a-based diagnostic tool was developed to detect the CHIKV genome. The *nsP1* regions of CHIKV were selected as target sites. Assay validation was performed using lateral-flow dipstick and fluorescence readouts.

## MATERIALS AND METHODS

### Clinical specimens

A total of 146 de-identified residual plasma samples from CHIKV-confirmed patients (collected 2019–2021) were obtained from Siriraj Hospital, Mahidol University. The study protocol was approved by the Siriraj Institutional Review Board, Mahidol University (COA 782/2022). The IRB waived the need for consent because of the anonymity of all specimens. All experiments were performed in compliance with relevant laws and institutional guidelines and in accordance with the ethical standards of the Declaration of Helsinki. The CHIKV (Ross strain) was obtained from the Thai National Institutes of Health (Thai NIH) and propagated in C6/36 cell line (ATCC: CRL-1660).

### CAS13a protein

Cas13a enzyme from *Leptotrichia wadei* (LwaCas13a) was prepared and stored according to our previously published protocol (15).

### Viral RNA extraction

CHIKV RNA from 146 samples was extracted using the MagLEAD 12gC automated extraction platform (Precision System Science, Chiba, Japan) according to the manufacturer’s instructions. Viral RNA was eluted with 100 µL of buffer and used for the RT-PCR assay and RPA-CRISPR-based assay. RNA extraction was performed using an automated platform to ensure consistent nucleic acid yield and purity across all samples. This standardized approach minimized variability that could arise from manual extraction and allowed the accurate assessment of the RPA-CRISPR assay’s analytical performance.

### Detection and quantification of CHIKV RNA

The Genesig Advanced CHIKV qRT-PCR Assay (Primerdesign Ltd., UK) and SuperScript III Platinum One-Step qRT-PCR Kit (Thermo Fisher Scientific, USA) were used to detect CHIKV RNA according to the manufacturer’s instructions. Briefly, 5 µL of extracted RNA was added to 10 µL of 2× Reaction Mix, 1 µL of SuperScript III RT/Platinum Taq Mix, and 1 µL of RNase-free water. A CFX-96 real-time thermal cycler (Bio-Rad Laboratories, Inc., Hercules, CA, USA) was used for amplification. The conditions consisted of 1 cycle of 5 min at 50°C and 2 min at 95°C, followed by 50 cycles of 10 s at 95°C and 10 s at 60°C. The results were obtained and recorded as cycle threshold (Ct) values for each sample. Quantification of CHIKV RNA was performed using digital droplet PCR (Bio-Rad Laboratories, Inc., Hercules, CA, USA) according to the manufacturer’s instructions. The assay used a primer set from the Genesig advanced CHIKV qRT-PCR assay (Primerdesign Ltd., UK).

### RPA primer and crRNA design

**Fig 1:**
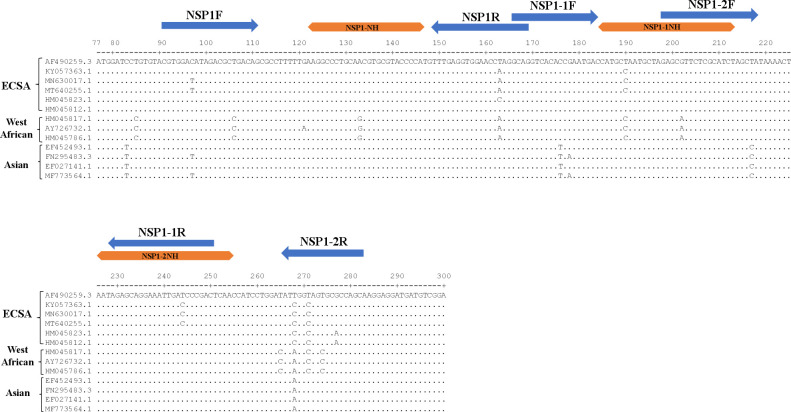
Alignment of the nsP1 gene of chikungunya virus, used for primer and crRNA design. The positions of forward and reverse primers are indicated with blue arrows, while crRNAs are marked with orange double-sided arrows. This study designed three sets of primer/crRNAs: set A (NSP1F/NSP1R/NSP1-NH), set B (NSP1-1F/NSP1-1R/NSP1-1NH), and set C (NSP1-2F/NSP1-2R/NSP1-2NH). The 13 sequences displayed represent a subset of the 50 complete sequences used for the initial *in silico* alignment and primer design. The nucleotide coordinates of nsP1 are referenced to the chikungunya virus Ross strain (GenBank accession AF490259).

To design RPA primers and crRNA, 50 complete CHIKV sequences covering the Asian, West African, and East/Central/South African genotypes were aligned using Lasergene Genomics Suite 12 (DNASTAR, Madison, WI, USA). The conserved region of the *nsP1* gene was selected as the target for RPA primer binding. The primers were designed using Primer3plus (https://www.primer3plus.com/index.html) with a length of approximately 20–25 nucleotides. The T7 promoter sequence was attached at the 5′ end of the forward RPA primer to facilitate the T7-based transcription of the resulting amplicon. The crRNAs were designed using the following criteria: a 36-nucleotide secondary structure loop for LwaCas13a recognition and binding and a 26–28-nucleotide protospacer complementary to the target sequence (19). Primer sets A, B, and C were designed to target CHIKV sequences of 79, 86, and 87 base pairs, respectively. The sizes of crRNA-A, crRNA-B, and crRNA-C were 26, 29, and 28 base pairs, respectively. The primers and crRNAs are shown in the alignment sequences in Fig. 1.

The primers and target sequences were mapped in the NCBI repository to test cross-reactivity with human host DNA and other viruses. The sequence of primers and crRNAs for *nsP1* is shown in Table 1. The primers and crRNAs were synthesized by Integrated DNA Technologies (Coralville, IA, USA).

**TABLE 1:** Comparison of clinical validation of SHERLOCK for chikungunya virus RNA detection by fluorescence and lateral flow

		Clinical validation samples tested by RT-qPCR (*N* = 146)		Performance characteristics			
		Positive	Negative	Sensitivity	Specificity	NPV	PPV
Fluorescence readout	Positive	71	0	97.3% (90.45%–99.67%)	100% (95.07%–100.00%)	97.3% (90.30%–99.31%)	100% (94.94%–100.00%)
	Negative	2	73				
	Total	73	73				
Lateral-flow readout	Positive	69	0	94.5% (86.56%–98.49%)	100% (95.07%–100.00%)	94.8% (87.56%–97.93%)	100% (94.79%–100.00%)
	Negative	4	73				
	Total	73	73				

### Recombinase polymerase amplification (RPA) reaction

A lyophilized RPA pellet (TwistAmp Basic kit; TwistDx, Cambridge, UK) was prepared by resuspending 29.5 µL of rehydration buffer into one pellet of RPA reaction. Volumes of 2 µL (10 mM each) of RPA primer mix, 1 µL (200 U/µL) of PrimeScript reverse transcriptase (Takara, Japan), 0.06 µL (60 U/µL) of RNaseH (Takara), and 0.66 µL (280 mM) of magnesium acetate were added to the RPA mixture. The mixture was aliquoted (6.51 µL) into a precooled reaction tube, and then 6 µL of CHIKV RNA template was added. The reactions were incubated at 42°C for 25 min and used in a subsequent step of the Cas13a detection reaction. Diethyl pyrocarbonate (DEPC)-treated water was used as a negative control in the RT-RPA reaction. This condition was used to optimize the performance of the assay by adjusting various parameters, such as primer concentrations, RPA reagent concentrations, and incubation times.

### Cas13a-based assay with a lateral flow readout

The Cas13a-based assay with a lateral-flow readout was performed as previously described (15). Briefly, a reaction mixture of 20 µL was prepared by adding 8.5 µL of DEPC-treated water, 2 µL of Tris (pH 7.4, 400 mM), 1 µL of SUPERase•In RNase Inhibitor (20 U/µL), 0.8 µL (25 mM) of ribonucleoside tri-phosphate mix (NEB, Ipswich, MA, USA), 0.28 µL (50 U/mL) of T7 RNA polymerase (NEB), 1 µL (20 µM) of FAM–Iowa Black PolyU reporter (Integrated DNA Technologies), 2 µL (63.3 ng/µL) of LwaCas13a enzyme in storage buffer, 1 µL (10 ng/µL) of crRNA, 1 µL (120 mM) of magnesium chloride, and 2 µL of RPA reaction product. The reaction mixture was incubated at 37°C for 30 min. Next, 80 µL of diluent HybriDetect assay buffer was added to the CRISPR13a assay mixture and then mixed. HybriDetect dipsticks (Milenia HybriDetect; Milenia Biotec, Giessen, Germany) were placed into mixture tubes, and the results were read within 7–10 min. The appearance of a band at the test line indicated a positive result, while a single band at the control line indicated a negative result.

### Cas13a-based assay with a fluorescence readout

To prepare Cas13a-based assay reactions, a mixture of 7.2 µL of DEPC-treated water, 2 µL of Tris (pH 7.4, 400 mM), 0.8 µL (25 mM) of ribonucleoside triphosphate mix (NEB), 0.6 µL (50 U/mL) of T7 RNA polymerase (NEB), 3.5 µL (2 µM) of FAM–Iowa Black PolyU reporter (Integrated DNA Technologies), 2 µL (63.3 ng/µL) of LwaCas13a enzyme in storage buffer, 1 µL (10 ng/µL) of crRNA, and 1 µL (120 mM) of magnesium chloride was added to a 1.5 mL tube and mixed. A volume of 18 µL of the Cas13a mixture was aliquoted into a PCR strip tube, followed by the addition of 2 µL of RPA product. The fluorescence signal was collected over 60 min at 1-minute intervals at 37°C on a CFX-96 real-time PCR system (Bio-Rad).

### Analytical performance of the Cas13a-based assay for detecting CHIKV

#### Limit of detection

A 10-fold serial dilution of CHIKV RNA was prepared and tested with the Cas13a assay with a lateral-flow or fluorescence readout. All assays were performed with three replicates for each dilution. The performance of three sets of forward and reverse RPA primers/crRNAs was evaluated using a fluorescence readout against CHIKV RNA at three different serial dilutions (10^−5^, 10^−6^, and 10^−7^). These dilutions were also quantified for CHIKV RNA using digital droplet PCR. After obtaining the lowest RNA concentration capable of producing positive signals, the dilution at the limit of detection (LOD) point was repeated with 10 further measurements. The lowest RNA concentration, using both lateral-flow and fluorescence readouts, was quantified by digital droplet PCR with Genesig primers and probes for CHIKV (Primerdesign).

#### Cross-reactivity

Cross-reactivity was evaluated using RNA from rubella virus (ATCC: VR315D) and flaviviruses (DENV1 (ATCC: VR-1856), DENV2 (ATCC: VR-1810), DENV3 (ATCC: VR-3380), DENV4 (ATCC: VR-1490), and ZIKV (ATCC: VR-1843). Rubella virus, DENV1-4, and ZIKV were purchased from the American Type Culture Collection (ATCC), while CHIKV (Ross strain) was obtained from the Department of Medical Science, Ministry of Public Health, Thailand.

### Clinical evaluation of the Cas13a-based assay

To assess the clinical performance of the Cas13a-based assay for detecting CHIKV, 175 leftover plasma samples were sent to a routine laboratory for CHIKV RNA detection. Total RNA was extracted from the samples, and CHIKV real-time PCR (Primerdesign Ltd.) was performed. Based on the Ct value, the specimens were categorized as follows: (i) a Ct value <20, (ii) a Ct value of 20–25.9, (iii) a Ct value of 26–29.9, (iv) a Ct value of 30–34.9, and (v) a Ct value >35

### Statistical analysis

The raw data from the fluorescence readout were analyzed using Prism GraphPad (Version 10; GraphPad Software, La Jolla, CA, USA). Comparisons between groups were performed using a one-way analysis of variance. The parameters representing clinical performance of the Cas13a-based assay, with a 95% confidence interval (CI) were calculated to determine sensitivity and specificity and were compared with real-time PCR.

## RESULTS

In this study, three RPA primer pairs were designed to target the *nsP1* gene specifically. The alignment analysis was conducted on reference CHIKV strains representing Western African, Asian, and Eastern/Central/Southern African (ECSA) genotypes. This study aimed to identify conserved regions that are universally shared among all genotypes. The alignment analysis showed the location of the primer pair targeting *nsP1* and the corresponding crRNA sequences specific to *nsP1* (Fig. 1). These primers amplified products of different sizes. Set A produced a 103-base pair product (targeted CHIKV size: 79 base pairs; crRNA-A size: 26 base pairs), set B produced a 110-base pair product (targeted CHIKV size: 86 base pairs; crRNA-B size: 29 base pairs), and set C produced a 111-base pair product (targeted CHIKV size: 87 base pairs; crRNA-A size: 28 base pairs).

### Optimization of Cas13-based detection of CHIKV

**Fig 2:**
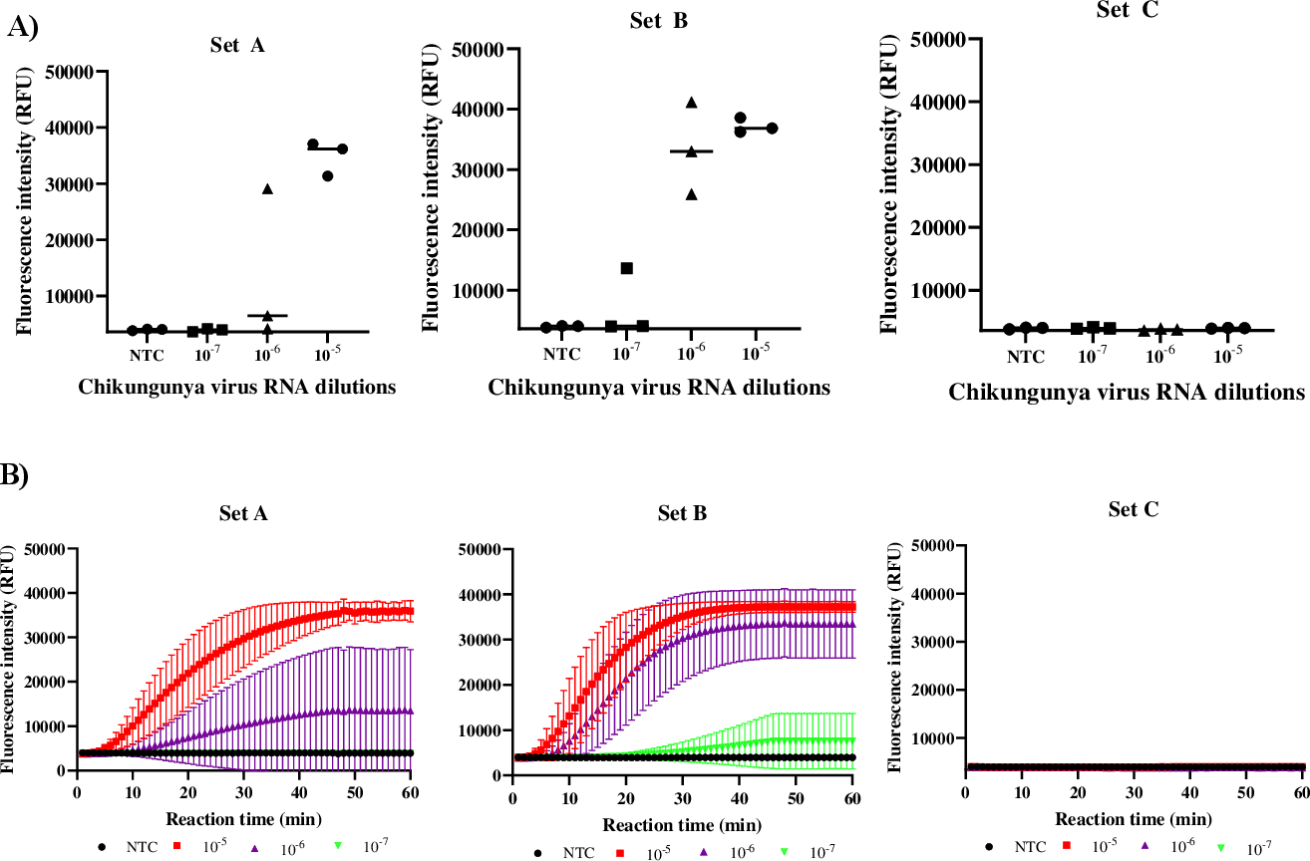
Evaluation efficiency of RPA primers and crRNA. (**A**) Endpoint fluorescence signal production over 60 min from three sets of RPA primers/crRNA against serial dilutions of chikungunya virus RNA. NTC, non-template control. (**B**) Fluorescence kinetic signal production over 60 min from three sets of RPA primers/crRNA against serial dilutions of chikungunya virus RNA.

**Fig 3:**
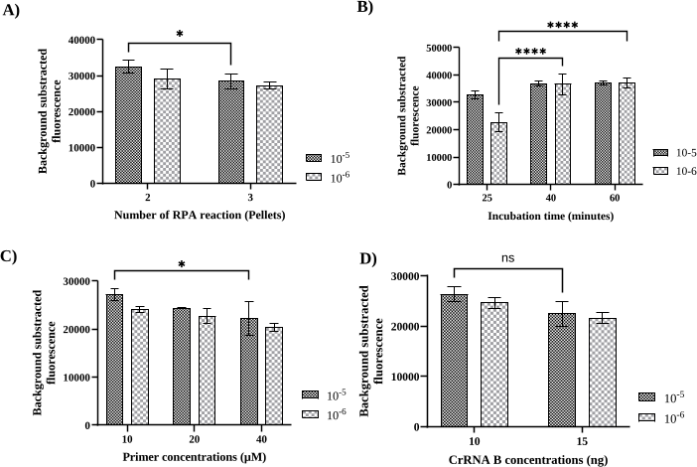
Optimization condition for RPA-CRISPR-based assay. The parameters for optimization included the concentration of RPA reaction (**A**) incubation time for RPA amplification (25, 40, and 60 min) (**B**), primer concentration of RPA assay (10, 20, and 40 µM) (**C**), and concentration of crRNA (10 and 15 ng/µL) (**D**). These were tested against chikungunya virus RNA at dilutions of 10^−5^ and 10^−6^ and measured using a fluorescence readout. The bar graph represents data of mean ± SD from three replicate experiments. Statistical significance: *P < 0.05; ****P < 0.0001; ns, not significant (*P* ≥ 0.05).

To determine the optimal RPA and CRISPR-based detection conditions, various parameters, such as primer pair selection, RPA conditions, and crRNA concentrations, were evaluated using fluorescence measurements. Three different serial dilutions (10^−5^, 10^−6^, and 10^−7^) of CHIKV RNA, corresponding to Ct values of 29.8 (equivalent to 2,469 copies/reaction), 34.0 (equivalent to 215 copies/reaction), and 36.9 (equivalent to 27 copies/reaction), respectively, were used to evaluate the performance of three sets of forward and reverse RPA primers/crRNAs. The primers specific to the *nsP1* region (set B) produced the highest fluorescence signals and could be used to detect the lowest copy number of CHIKV RNA compared with the other sets (Fig. 2A and B). Once the RPA primer and crRNA set had been selected, the performance of the other parameters was evaluated against CHIKV RNA at 10^−5^ and 10^−6^ dilutions using the fluorescence readout. The first parameter evaluated was the amount of RPA reagent used. Two versus three freeze-dried RPA pellets in resuspension buffer to produce a reaction mixture for 10 reactions were examined for detecting CHIKV. Using two pellets for 10 reactions showed higher fluorescence signals than the three-pellet condition, with a significant difference in signal upon using a Ct value of 29.8 RNA input (*P* < 0.05, Fig. 3A). Therefore, two RPA pellets for 10 reactions were used for all further studies. The incubation time of the RPA reaction was then varied between 25, 40, and 60 min. The incubation times of 40 min and 60 min showed higher fluorescence signals than the 25-minute condition when using a Ct value 34.0 (*P* < 0.05, Fig. 3B). There was no significant difference in fluorescence signals between the 40- and 60-min reaction times. Therefore, we chose the shorter time point of 40 min for further evaluation. The concentration of RPA primers was also evaluated by varying them between 10, 20, and 40 µM. Although not statistically significant, the 10 µM concentration showed a slightly higher signal for detection than the 20 µM concentration (*P* > 0.05) (Fig. 3C). The last parameter evaluated for optimization was crRNA concentrations, which varied between 10 and 15 ng/µL. Similarly, although not statistically significant, the concentration of 10 ng/µL crRNA showed a slightly better signal than 15 ng/µL (*P* > 0.05) (Fig. 3D). Therefore, 10 ng/µL crRNA was used for further evaluation.

### Assay validation

#### LOD and cross-reactivity

**Fig 4:**
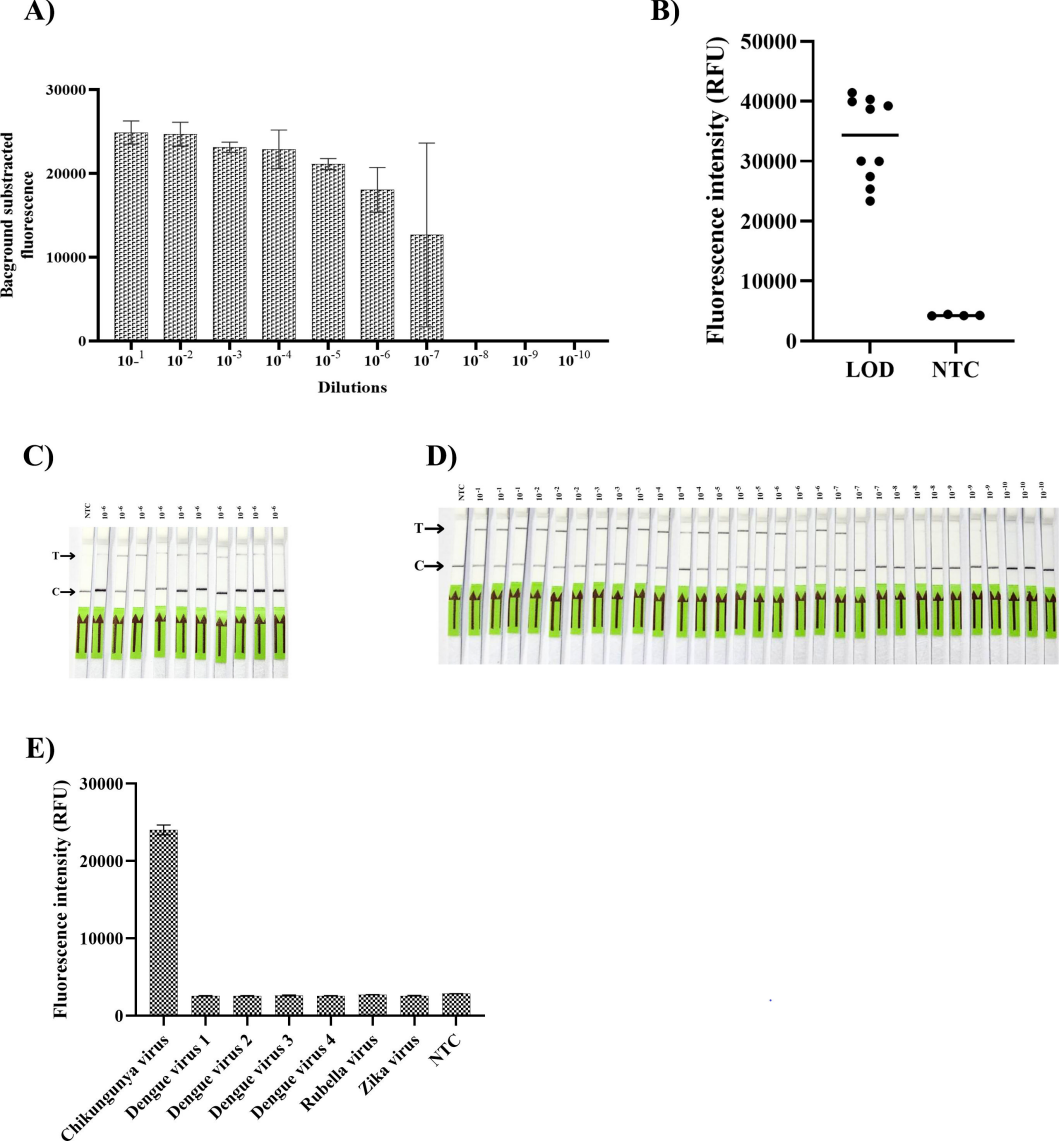
Analytical sensitivity and specificity of the CRISPR-Cas13a assay for chikungunya virus detection. Sensitivity of chikungunya SHERLOCK was determined by serial ten-fold dilutions of chikungunya virus RNA, which was performed and detected in three replicates with RPA-CRISPR assay. The limit of detection (LOD) was evaluated by (**A**) fluorescence readout and (**B**) lateral-flow readout. Ten measurements at LOD values were performed and demonstrated in (**C**) fluorescence readout and (**D**) lateral-flow readout. (**E**) The specificity of the assay was assessed against seven viruses, including chikungunya virus, dengue virus types 1–4, Zika virus, and rubella virus.

**Fig 5:**
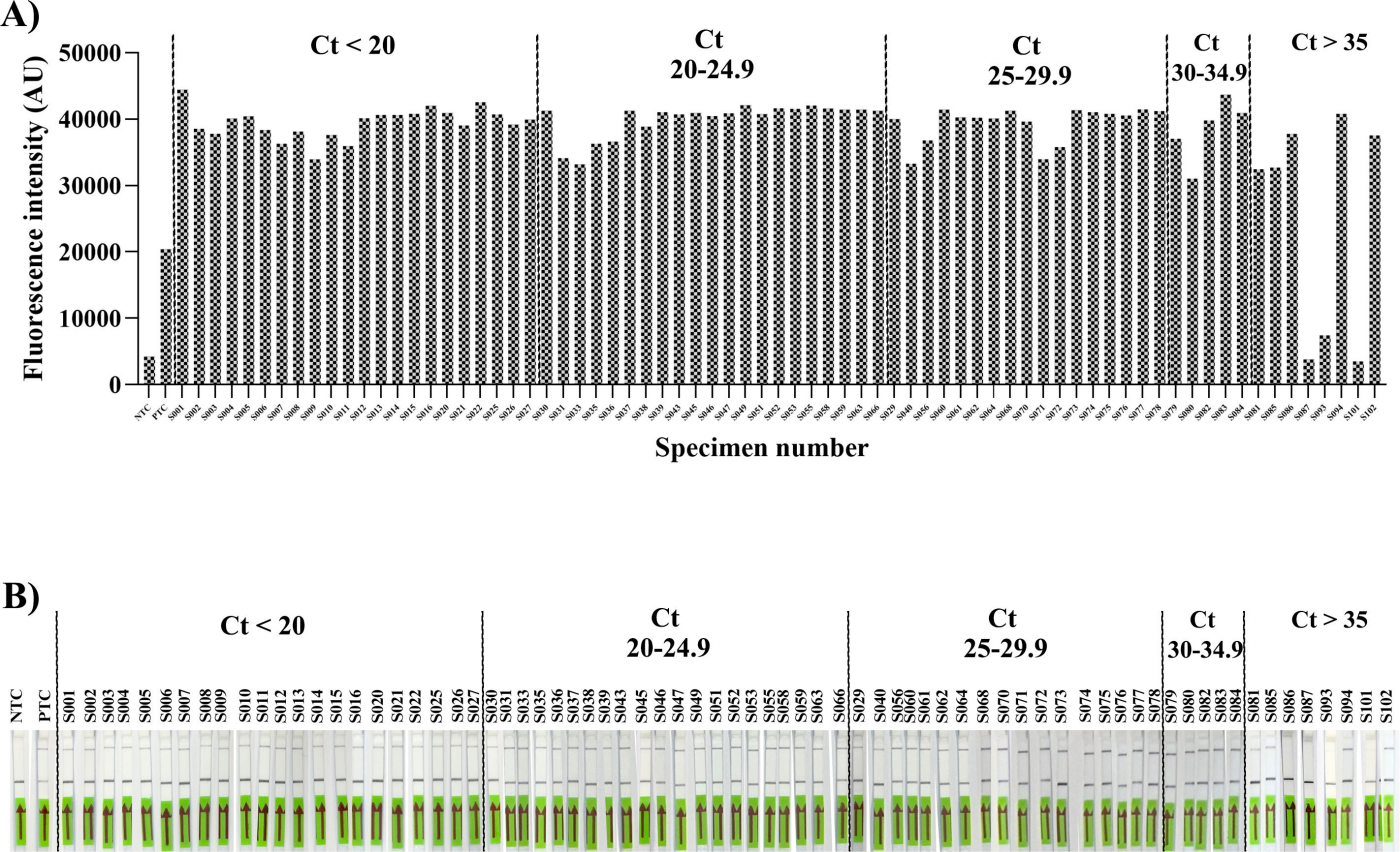
Clinical performance of RPA-CRISPR/Cas13a for chikungunya virus detection. Positive chikungunya virus samples with varying Ct values (Ct <20, Ct 20–24.9, Ct 25–29.9, Ct 30–34.9, and Ct >35) were detected with (**A**) fluorescence readout and (**B**) lateral-flow readout. NTC and PTC are non-template control and positive control. On a lateral-flow assay, C and T refer to the control line and test line, respectively.

To assess the LOD of the Cas13-based assay for CHIKV, a serial dilution of known concentrations of extracted RNA from CHIKV cultivated in C6/36 cells was used in the assay, and the results were analyzed using lateral-flow and fluorescence readouts. The best conditions of the four parameters (RPA concentration [two pellets/10 reactions], RPA primer concentration [10 µM], RPA incubation time [40 min], and crRNA concentration [10 ng/µL]) from the optimization steps were used to evaluate the LOD. The fluorescence signal was detected up to a dilution of 10^−7^, which corresponded to a Ct value of 36.9 (27 copies/reaction) (Fig. 4A). However, this dilution was detected in only two of the three reactions. The lateral-flow readout showed similar results to fluorescence, except that at a 10^−7^ dilution, it could detect only one of three reactions (Fig. 4A and D). Therefore, the 10^−6^ dilution (corresponding to a Ct value of 34.0 [215 copies/reaction]) was established as the LOD for this experiment. To confirm the LOD value, 10 replicates at a 10^−6^ dilution (215 copies/reaction, equivalent to 10.7 copies/mL) were performed (Fig. 4B and C). The signal was detected in all replicates by fluorescence and lateral-flow readouts (Fig. 4B and C). SHERLOCK targeting of the *nsP1* gene showed high specificity for CHIKV, with no cross-reactivity to rubella virus or flaviviruses tested (Fig. 5).

#### Clinical evaluation of Cas13-based detection of CHIKV

To validate the assay against the clinically relevant strain responsible for recent regional outbreaks, all performance characteristics were determined using the ECSA lineage. To assess the performance of the CRISPR-based assay for detecting CHIKV, 146 plasma samples were used for validation. Of these, 73 samples were positive, and 73 samples were negative by qPCR. All samples were subjected to rigorous blinding protocols and subsequently dispatched to the experimenters for analysis. Lateral-flow and fluorescence detection were performed in parallel. The Ct value of CHIKV-positive qPCR from 73 samples ranged from 16.29 to 37.03. Among the positive samples, 69 of 73 (94.52%) samples were detected using the lateral-flow readout, while 71 of 73 (97.26%) samples were detected using the fluorescence readout. All negative samples by qPCR showed no intense test band in the lateral-flow readout and no signal for the fluorescence readout. Among the positive samples, Group 1, composed of 18 samples with a Ct value of <20, demonstrated 100% detection by both readouts. Similarly, Group 2 (25 samples with Ct values between 20 and 24.9), Group 3 (17 samples with Ct values between 25 and 29.9), and Group 4 (5 samples with Ct values between 30 and 34.9) each demonstrated 100% detection by both readouts. Group 6, consisting of eight samples with Ct values >35, showed 37.5% detection (3/8, with Ct values of 35.7, 35.97, and 36.41) by both readouts, while another 37.5% (3/8, with Ct values of 36.55, 36.61, and 37.03) were detected only by the fluorescence readout. The remaining two samples showed no band or signal on either readout (Ct values of 36.59 and 36.93). Therefore, the total clinical sensitivity, specificity, positive predictive value, and negative predictive value of the lateral-flow readout were 94.52 (95% CI, 90.45%–99.67%), 100% (95.07%–100.00%), 100% (94.79%–100.00%), and 94.81% (87.56%–97.93%), respectively. In addition, the clinical sensitivity, specificity, positive predictive value, and negative predictive value of the fluorescence readout were 97.26% (95% CI, 90.45%–99.67%), 100% (95.07%–100.00%), 100% (94.94%-100.00%), and 97.33% (90.30%–99.31%), respectively (Fig. 5).

## DISCUSSION

CHIKV causes an annual outbreak in Thailand during the monsoon season. The morbidity rate of CHIKV increased in 2009 (82.03/100,000 population) and has since decreased (20). In 2023, the morbidity rate from CHIKV infection in Thailand was 2.15/100,000 population (21). Early identification of CHIKV is critical for controlling viral spread and initiating effective therapeutic management. Laboratory assays for CHIKV detection rely on antigen and antibody detection, including rapid antigen/antibody and ELISA. Antigen detection shows a high sensitivity in the acute phase (fever <7 days). The sensitivity of rapid and ELISA-based antigens was 85.8% and 82.2%, respectively (22). However, antibody detection (immunoglobulin M) showed the lowest sensitivity (42.3%), particularly in the rapid-test format, compared with ELISA (93.4%) (22). A molecular diagnostic test showed high sensitivity (98.4%) for detecting CHIKV (23). However, molecular methods require specialized equipment, particularly a real-time PCR machine, which is expensive and often unaffordable in resource-limited settings. A low-cost alternative diagnostic tool with equivalent sensitivity to real-time PCR is required in resource-limited settings. During the outbreak of SARS-CoV-2, the requirement for rapid diagnosis to control infection prompted the development and validation of isothermal amplification techniques, such as RPA and the loop-mediated isothermal amplification/CRISPR-based system, as alternative methods (15, 24). The authors previously successfully developed an RPA/CRISPR-based method for rapidly detecting SARS-CoV-2 with high sensitivity (15). The RPA/CRISPR-based system has been applied to detect many pathogens, such as dengue virus and Zika virus, and was recently applied to CHIKV (24, 25). The current study used RPA coupled with a CRISPR-Cas13a-based system, which was adapted from a previous protocol, to detect the CHIKV genome. Moreover, this CRISPR-Cas13a-based assay uses the eye visualization method, which is suitable for resource-limiting settings.

Molecular detection of the target region of CHIKV usually relies on two genes, namely *nsP1* and *E1* (22, 26, 27). Because of the variability of *E1*, this region is commonly used for genetic diversity studies (28), while the *nsP1* gene is a common target for the molecular diagnosis of CHIKV (26). Therefore, the current study aimed to use the *nsP1* gene as a target for detecting CHIKV RNA with the CRISPR-Cas13a-based assay. The RPA-coupled CRISPR-Cas13a assay begins with nucleic acid extraction, followed by two subsequent steps of RPA amplification and signal detection using CRISPR-Cas13a detection. To design the RPA primers and crRNAs in this study, alignment of the *nsP1* gene from CHIKV reference strains was performed, and a conserved region between the strains for the RPA primers and crRNAs was selected. Three sets of primers/crRNAs were designed and used to evaluate their performance in the amplification and detection steps. The evaluation results showed that the primers and crRNAs of sets A and B showed signals after detection. However, the primers and crRNAs of set B showed a higher signal and were able to detect low copy numbers of CHIKV RNA, as observed with a kinetic curve, compared with set A. To set up an RPA-CRISPR-based assay for a diagnostic test, several parameters must be optimized, such as RPA reaction concentrations, the RPA incubation time, and primer and crRNA concentrations. The optimal conditions for this study were one pellet of RPA for five reactions, 10 µM of primer, 10 ng/ µL of crRNA, and an RPA amplification time of 40 min. This condition was set and used for the establishment of the LOD. A series of 10-fold dilutions of CHIKV RNA was used to detect the signals by fluorescence and lateral flow. The LOD of this assay was 215 copies/reaction (corresponding to a Ct value of 34). Although the fluorescence readout detected a signal in two of three replicates and the lateral flow detected a signal in one of three replicates at 27 copies/reaction (corresponding to a Ct value of 36.9), we could not accept this dilution as the LOD for this assay because it showed less than 95% reliability. The LOD of this assay was confirmed by performing 10 replicates at this dilution, and the assay was able to detect all replicates using both methods. Therefore, the LOD of this assay was 215 copies/reaction (10.7 copies/µL), which showed a potential to detect CHIKV during the acute phase (1–4 days of illness), when viral loads typically range from 1.7 × 10^2^ to 9.9 × 10^6^ copies/µL (29). Moreover, our developed assay was specific to CHIKV and was tested against closely related members of the flavivirus family.

Our clinical performance evaluation using 146 plasma samples showed an assay sensitivity of 97.26% and a specificity of 100% with the fluorescence readout. Similarly, with the lateral-flow readout, we observed a sensitivity of 94.52% and a specificity of 100%. Two positive samples were sequenced and identified as belonging to the ECSA lineage responsible for the outbreak in Thailand (GenBank accession numbers: PV066168 and PV066169), confirming that the developed assay can reliably detect this circulating strain. In contrast to a previous method that used isothermal amplification to detect CHIKV RNA, the assay in this study showed a lower sensitivity (97.26%) than the previous study (100%) (30). However, the specificity of the assay in the current study was higher than that in another previous study (96.72%) (30). In addition, a previous study using CRISPR-Cas12a for detecting CHIKV showed 100% sensitivity, which is higher than that observed in the current study (25). The missed detection of positive samples with late Ct values contributed to the lower sensitivity of the current results. However, because the previous study did not report Ct values for their samples, definitively concluding that the clinical sensitivity in the previous study was indeed higher than that in the current study is difficult (25). Compared with the Cas12a-based one-pot assays described by Bhardwaj et al. and Broughton et al.*,* our Cas13a-based SHERLOCK platform introduces key methodological improvements. Unlike Cas12a systems, which rely on DNA intermediates and one-pot reactions, Cas13a directly targets RNA, reducing amplification bias and enabling faster, highly sensitive detection. The two-step RPA-Cas13a workflow also allows better control of amplification and collateral cleavage kinetics, thereby improving reproducibility and analytical precision. Furthermore, our assay was validated using a larger and clinically diverse plasma cohort (*n* = 146) and evaluated by both fluorescence and lateral-flow readouts, demonstrating high diagnostic accuracy and adaptability for field deployment. Collectively, these distinctions highlight the enhanced flexibility and RNA-specific advantages of the Cas13a SHERLOCK system compared with existing Cas12a-based assays (26, 27). Moreover, when the clinical sensitivity was recalculated using a Ct value cutoff based on the assay’s LOD (Ct value of 34), 100% sensitivity was observed within this cutoff for both readouts. The total time for detecting CHIKV was 80 min, starting with the extraction step. The CRISPR-Cas13a assay demonstrated high analytical and clinical performance; however, the current workflow still relies on laboratory-based equipment, including automated RNA extraction, temperature-controlled incubation, and fluorescence detection. These requirements prevent its immediate deployment as a true point-of-care (POC) test. Future work will focus on simplifying the extraction process and integrating portable detection modules to enhance field applicability. Specifically, while fluorescence-based detection offers higher sensitivity and quantitative capability, lateral-flow readout provides a simpler and more field-adaptable alternative requiring minimal equipment and only basic incubation conditions. The choice of detection platform should therefore balance analytical performance with logistical feasibility, depending on local resource availability. Although the *in silico* analysis suggests that the chosen target region is conserved across major CHIKV genotypes, the empirical validation presented here was limited to the ECSA lineage. Further studies are required to confirm equivalent performance against other globally significant lineages, such as the Asian and West African lineages. While the CRISPR-Cas13a assay demonstrated high analytical and clinical performance, the current workflow still relies on laboratory-based equipment, including automated RNA extraction and temperature-controlled devices. This dependence limits its immediate deployment as a true point-of-care diagnostic. Future work will focus on integrating simplified extraction and visual detection modules to enhance portability and field applicability.

One limitation of the developed assay is the absence of an internal control to check for false-negative results caused by inhibitors. In the future, if this system is adapted into a point-of-care device, all quality control measures will be thoroughly addressed. Second, the developed assay has not been tested by adding plasma directly to the system. This process will decrease the detection time if the RNA extraction process can be omitted. Third, we acknowledge that the comparison of extraction methods to assess point-of-care feasibility could not be performed in this study. Most archived clinical specimens were fully consumed during the initial testing and validation process, preventing re-extraction. Nevertheless, a comparative evaluation between automated and simplified extraction workflows would be valuable for future implementation studies. Additionally, this study was limited to the validation of the ECSA lineage due to the unavailability of other lineage-specific viral materials or RNA standards. Further evaluation across diverse CHIKV lineages will be important to confirm the assay’s analytical sensitivity and cross-lineage performance. Moreover, the clinical validation was based on samples collected from a single hospital between 2019 and 2021, which may limit the representation of geographic and genotypic diversity. This limitation reflects the availability of archived, well-characterized clinical specimens within the project’s timeframe and biosafety constraints. Future work should therefore include multicenter sample collection and incorporate recent CHIKV strains to enhance the generalizability and lineage coverage of the assay. Finally, although testing for cross-reactivity with other alphaviruses, such as O’nyong-nyong virus (ONNV), Mayaro virus (MAYV), and Ross River virus (RRV), would further strengthen the assay’s analytical specificity, these viruses are not known to circulate in Thailand or neighboring Southeast Asian countries. Thus, corresponding viral materials were unavailable for this study.

In summary, the CRISPR-Cas13-based system has the potential to detect CHIKV RNA with high sensitivity and specificity. Therefore, this tool could be useful for the diagnosis of CHIKV, particularly in resource-limited settings.

## Data Availability

All nucleotide sequences generated in this study have been deposited in GenBank under accession numbers PV066168 and PV066169.
